# Untangling Tau and Iron: Exploring the Interaction Between Iron and Tau in Neurodegeneration

**DOI:** 10.3389/fnmol.2018.00276

**Published:** 2018-08-17

**Authors:** Shalini S. Rao, Paul Anthony Adlard

**Affiliations:** Division of Mental Health, The Florey Institute of Neuroscience and Mental Health, The University of Melbourne, Parkville, VIC, Australia

**Keywords:** tau, iron, Alzheimer’s disease, metal, deferiprone

## Abstract

There is an emerging link between the accumulation of iron in the brain and abnormal tau pathology in a number of neurodegenerative disorders, such as Alzheimer’s disease (AD). Studies have demonstrated that iron can regulate tau phosphorylation by inducing the activity of multiple kinases that promote tau hyperphosphorylation and potentially also by impacting protein phosphatase 2A activity. Iron is also reported to induce the aggregation of hyperphosphorylated tau, possibly through a direct interaction via a putative iron binding motif in the tau protein, facilitating the formation of neurofibrillary tangles (NFTs). Furthermore, in human studies high levels of iron have been reported to co-localize with tau in NFT-bearing neurons. These data, together with our own work showing that tau has a role in mediating cellular iron efflux, provide evidence supporting a critical tau:iron interaction that may impact both the symptomatic presentation and the progression of disease. Importantly, this may also have relevance for therapeutic directions, and indeed, the use of iron chelators such as deferiprone and deferoxamine have been reported to alleviate the phenotypes, reduce phosphorylated tau levels and stabilize iron regulation in various animal models. As these compounds are also moving towards clinical translation, then it is imperative that we understand the intersection between iron and tau in neurodegeneration. In this article, we provide an overview of the key pathological and biochemical interactions between tau and iron. We also review the role of iron and tau in disease pathology and the potential of metal-based therapies for tauopathies.

## Introduction

Neurofibrillary tangles (NFTs) are a pathological hallmark of a class of disorders known as tauopathies, that includes Alzheimer’s disease (AD), progressive supranuclear palsy (PSP), Parkinson’s disease (PD), Huntington’s disease (HD), Pick’s disease (PiD) and frontotemporal dementia with parkinsonism-17 (FTDP-17; Goedert et al., 2012). The primary constituent of NFTs is paired helical filaments (PHFs) composed of the hyperphosphorylated tau protein, an intrinsically unfolded and highly soluble phosphoprotein that is encoded by the *MAPT* gene (Weingarten et al., 1975; Spillantini and Goedert, 2013). Over the years, *in vitro* and *in vivo* studies have identified novel functions of tau in both normal physiology and disease, with reported roles in axonal transport, protein trafficking, cognitive function (Wang and Mandelkow, 2016) and interactions with proteins such as α-synuclein (the main component of Lewy Bodies (LBs) in PD; Lei et al., 2010) and amyloid-ß (Aß; the primary constituent of plaques found in AD; Bloom, 2014). Furthermore, it is well established that mutations in the *MAPT* gene are the primary cause of disease in FTDP-17 patients (Hutton et al., 1998). Whilst much is known about the regulation of tau, such as the effects of various kinases and phosphatases on the phosphorylation state of the protein which can then lead to conformational changes in protein structure (Zhu et al., 2015), a loss of tau function and the subsequent formation of PHFs and NFTs (Morris et al., 2011), there remains much to learn about this protein and the mechanisms through which it can influence both healthy and pathological aging. One such example is the emerging interaction between tau and iron. Dysregulation in cerebral iron is reported to be associated with the progression of tau-mediated neurodegeneration, with elevations in iron in affected brain regions that correlate both with the progression of neurodegeneration and the formation of NFTs in tauopathies (Andrasi et al., 1995; Duce et al., 2010). Further to this, reducing the burden of iron has been shown to alleviate behavioral deficits and reduce the formation of NFTs in animal models (Fine et al., 2012; Prasanthi et al., 2012; Guo et al., 2013a). In this review, we will provide a brief overview of the structure and function of tau and also iron metabolism, as these topics have been reviewed extensively. The main scope of this article is to revise the role of iron and tau in disease, to examine the proposed interaction between iron and tau and to review the potential of metal chelation as a therapeutic strategy for tauopathies.

### Tau Physiology

**Figure 1:**
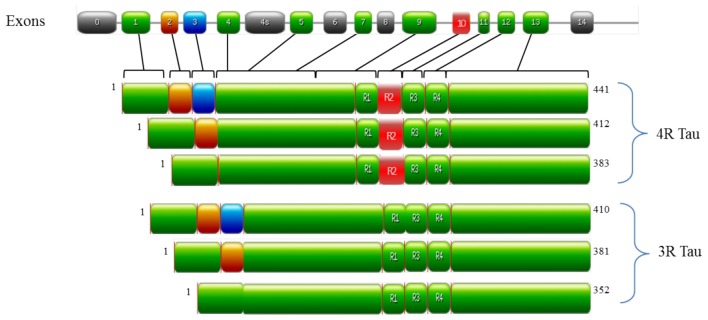
Structure of MAPT and the six tau isoforms expressed in an adult human brain. MAPT consists of 16 exons (E). Alternative mRNA splicing of E2 (orange), E3 (blue) and E10 (red) generates six tau isoforms ranging from 352–441 amino acids, The alternative splicing of additional exons (E1, E4, E5, E7, E9, E11, E12 and E13) are shown in green. E0, is part of the promoter and E14 are non-coding (gray). E6 and E8 (gray) are not transcribed in human brain. E4a (gray) is expressed only in the peripheral nervous system. The repeats motifs of tau (R1–R4) are shown, with three isoforms having four repeats each (4R Tau) and three isoforms having three repeats each (3R tau). Each repeat is 31 or 32 amino acids in length. Image was generated with PROSITE (Hulo et al., 2008).

In the human brain, tau is encoded by the *MAPT* gene on chromosome 17q21 and is predominately localized in the axons of neurons and to a lesser extent in dendrites and glial cells (Binder et al., 1985). The alternative splicing of *MAPT* generates six tau isoforms (as outlined in Figure 1), which differ by one or two 29 amino acid N-terminal inserts and by the number of microtubule binding domains (MBD; either 3 or 4, referred to as either 3R or 4R tau respectively; Goedert et al., 1989a,b; Andreadis et al., 1992). Under physiological conditions, 3R and 4R tau isoforms are expressed equally in most regions of the brain (Goedert and Jakes, 1990). However, alterations in this ratio (normally an increase in 4R tau) are observed in FTD-17 and PSP (Goode et al., 2000). Tau is unusually hydrophilic; as a phosphoprotein the longest tau isoform contains 84 putative phosphorylation sites, which includes 45 serines, 35 threonines and 4 tyrosines (Goedert et al., 1993; Chen et al., 2004; Götz et al., 2010; Mandelkow and Mandelkow, 2012). The structure of tau is subdivided into three domains, all of which are important in facilitating the interaction of tau with microtubules: (i) the projection domain located in the N-terminal region, determines the spacing between microtubules; (ii) the proline rich domain, comprised of up to seven PXXP motifs that are mainly serine-proline (SP) or threonine-proline (TP) motifs (targets of proline-directed kinases); and (iii) the assembly domain, located in the C-terminal half of the protein and consisting of the MBD, which interacts directly with microtubules (Weingarten et al., 1975; Cleveland et al., 1977; Steiner et al., 1990; Chen et al., 1992; Gustke et al., 1994; Lee et al., 1998). The assembly domain is of particular importance in understanding tau pathology as this region can seed and form PHFs (Pérez et al., 1996; Arrasate et al., 1999; Mukrasch et al., 2005). This characteristic of tau is quite counterintuitive to its physiological nature as tau is natively unfolded in solution and has low tendency for aggregation, as evidenced by various biophysical methods such as circular dichroism (CD; Schweers et al., 1994), nuclear magnetic resonance (NMR; Mukrasch et al., 2009) and small angle X-ray scattering (Mylonas et al., 2008). The MBD is also reported to interact with other proteins such as α-synuclein (Jensen et al., 1999), apolipoprotein E (ApoE; Strittmatter et al., 1994; Huang et al., 1995) and presenilin 1 (Takashima et al., 1998b). Though the function of tau is well established in microtubule stabilization and regulation, it is also involved in regulating axonal transport by interacting with p150, the major component of the dynein–activator complex dynactin (Magnani et al., 2007; Dixit et al., 2008). Further studies speculate a role for tau in neurotransmission via the Fyn pathway (Stamer et al., 2002; Ittner et al., 2010; Lei et al., 2012) and iron metabolism by the trafficking of APP (Lei et al., 2012). Tau function is regulated by its degree of phosphorylation, which modulates the binding of tau to microtubules and axonal transport (Sengupta et al., 1998; Abraha et al., 2000; Alonso Adel et al., 2004; Haase et al., 2004; Cuchillo-Ibanez et al., 2008). For example, phosphorylation of tau at Ser262, Thr231 and Ser235 inhibits its binding to microtubules by 35%, 25% and 10%, respectively (Sengupta et al., 1998), whilst phosphorylation at Thr231, Ser396 and Ser422 promotes aggregation of tau into filaments (Alonso Adel et al., 2004). As ~50% of tau phosphorylation sites are followed by proline; kinases of the proline-directed protein kinases (PDPK) family play a significant role in tau phosphorylation. The main PDPKs of tau include glycogen synthase kinase 3ß (GSK3ß), cyclin dependent kinase-5 (CDK5) and mitogen-activated protein kinases (MAPK). Phosphorylation sites not followed by proline are phosphorylated by non-PDPKs, include protein kinase A (PKA) and microtubule affinity regulating kinases (MARK; Dolan and Johnson, 2010). Tau phosphorylation sites are subject to the activity of more than one kinase as a form on inter-regulation between kinases (Martin et al., 2013). Indeed, a combination of PDPKs and non-PDPKS work synergistically to modulate tau phosphorylation under both physiological and pathological conditions (Martin et al., 2013). Candidate phosphatases of tau include serine/threonine protein phosphatase PP1, PP2A and PP2B (Hanger et al., 2009). PP2A is the main regulator of tau phosphorylation, which accounts for ~70% of tau phosphatase activity in the brain (Goedert et al., 1995; Wang et al., 1995, 1996). In a similar manner, PP2A activity is regulated by Pin 1, a phosphorylation dependent cis/trans isomerase which interacts with SP and TP motifs to regulate tau dephosphorylation (Liou et al., 2003). Though tau undergoes several post-translational modifications such as glycation, ubiquitination and truncation (Gong et al., 2005); aggregates of hyperphosphorylated tau are the primary components of NFTs and this pathway has been studied extensively in understanding tau dysregulation.

## Tau Pathology

Whilst the etiology of tau-mediated neurodegeneration is still unknown, it is generally accepted that tau hyperphosphorylation is one the key steps in disease progression which may lead to a “loss of tau function” and/or “gain of toxic function.” In familial tauopathies, MAPT mutations appear to be the primary cause of tau mediated neurodegeneration. However, in the case of sporadic tauopathies, the events or processes which trigger degeneration remain elusive.

### Tau Mutations

The most direct cause and effect link is established between* MAPT* mutations and the development of FTDP-17, PSP HD and corticobasal degeneration (CBD; Clark et al., 1998; Coppola et al., 2012; Kouri et al., 2014). To date there are 80 mutations, of which 51 are missense mutations (that alter sequencing or splicing of tau), located within exons 9 to 13 of *MAPT*. A majority of the missense mutations are clustered around the MBD of tau and effectively reduce the binding affinity of tau to microtubules (such as G272V, P301L/S, V337M, G389R and R406W; Hasegawa et al., 1998; Hong et al., 1998) and potentially retard axonal transport (Zhang et al., 2004; Gilley et al., 2012). Furthermore, these mutations are reported to generate tau mutants more prone to aggregation (Alonso Adel et al., 2004), possibly by increasing the amount of free soluble tau (unbound tau), which is a favorable substrate for hyperphosphorylation (Sengupta et al., 2006). Other mutations cause a change in the 3R:4R tau ratio (N279K, ΔN296, N296N, N296H, S305N and S305S)—and this is typically an increase in 4R-tau (Hong et al., 1998; Delisle et al., 1999; Hasegawa et al., 1999; Spillantini et al., 2000; Stanford et al., 2000; Iseki et al., 2001). Despite the heterogeneity of tauopathies caused by *MAPT* mutations, they all appear to reflect the burden of tau pathology and cause degeneration in brain regions known to serve specific cognitive, executive or motor functions. The research linking MAPT mutations to the development of tauopathies has led to the generation of animal models over-expressing tau mutations which develop phenotypes and biochemical changes associated with tauopathies. As such, they are valuable tools in understanding the mechanisms of tau-mediated neurodegeneration.

### A Balancing Act: Tau Hyperphosphorylation

Tau is highly phosphorylated in the later stages of neurodegeneration, which is speculated to be a result of an imbalance between kinase and phosphatase activity. Normal tau contains 2–3 moles of phosphate per mole of tau (Ksiezak-Reding et al., 1992), which is significantly increased in a range of human tauopathies (Ksiezak-Reding et al., 1992; Wray et al., 2008). As a majority of tau phosphorylation sites are followed by proline residues (Martin et al., 2011), it is not surprising that the activity of PDPK kinases such as GSK3β, CDK5 and MAPK are strongly linked to development of tau-mediated neurodegeneration. Indeed, these kinases are found to be associated with early tau deposits and tangles in brain samples from PSP, Pick’s Disease and AD (Pei et al., 1998; Perry et al., 1999; Ferrer et al., 2001a,b, 2002). Overexpression of GSK3β and the CDK-5 regulatory protein p25 in transgenic mouse models causes spatial learning deficits correlating with increased tau phosphorylation (Patrick et al., 1999; Lucas et al., 2001; Hernández et al., 2002; Noble et al., 2003; Li et al., 2004; de Barreda et al., 2010; Gómez de Barreda et al., 2010). Conversely suppression of tau kinases prevents tau hyperphosphorylation and neurodegeneration (Hong et al., 1997; Engel et al., 2006). These studies provide strong evidence for the aberrant activation of tau kinases in tauopathies. In addition, PP2A may drive tau hyperphosphorylation, as its activity is significantly compromised in AD brains and animal models of FTD (Liu et al., 2005; Tanimukai et al., 2005; Khandelwal et al., 2012). Tau dephosphorylation is achieved through the direct binding of PP2A to the MBD of tau (Sontag et al., 1999; Xu et al., 2008), as its hyperphosphorylation leads to a conformational change in its structure, this may prevent the binding of PP2A to tau. Furthermore, several *MAPT* mutations such as P301L and R406W are reported to reduce the binding affinity of PP2A to tau (Goedert et al., 2000). As a heterotrimeric holoenzyme, PP2A is comprised of a scaffolding A subunit, a variable regulatory B subunit and a catalytic C subunit. The activity of PP2A can be inhibited by the phosphorylation of the catalytic subunit at tyrosine-307 (Tyr307). Phosphorylated PP2A is found to co-localize with tangles in both AD and PD brains (Liu et al., 2008; Arif et al., 2014). The dysregulation of PP2A is also reported to modulate the activity of tau kinases mentioned above, both *in vivo* and *in vitro* (Pei et al., 2003; Wang et al., 2010; Louis et al., 2011). For example, PP2A dysregulation upregulates GSK3β activity, which may participate in inhibitory phosphorylation of PP2A (Yao et al., 2011). Conversely, the inhibition of GSK3β reverses PP2A inhibition and reduces phosphorylated tau levels *in vitro* (Martin et al., 2009). The phosphatase is also regulated by methylation; removal of the methyl group inactivates PP2A (Xing et al., 2008), which is also evidenced in AD brains (Sontag et al., 2004a,b). Dysregulation of methylated PP2A is also reported to be implicated in APP processing (Sontag et al., 2007); demethylated PP2A is found to drive Aβ induced neurotoxicity *in vivo*, whereas regulated PP2A methylation protected against neurodegeneration (Nicholls et al., 2016).

### Tangled

Neurodegeneration attributed solely to tau dysfunction such as in PSP may be driven by *MAPT* mutations and dysregulation in tau phosphorylation. However, in other tauopathies such as AD and PD, neurodegeneration may be mediated through interactions with key disease-relevant proteins such as ApoE4, APP or α-synuclein. Senile plaques (comprised of Aβ) and NFTs are the hallmark pathology of AD, though the accumulation of plaques does not correlate with age-dependent cognitive impairment, NFT formation on the other hand strongly correlates with brain degeneration and cognitive function (Wilcock and Esiri, 1982; Arriagada et al., 1992; Itoh et al., 1996; Perez-Nievas et al., 2013). It is suggested, however, that Aβ may drive tau-mediated neurodegeneration. Injection of synthetic Aβ in brains of transgenic mice overexpressing the tau P301L mutation, increases NFT formation 5-fold in regions near the injection sites (Götz et al., 2001). Furthermore, Aβ deposits develop prior to tangle pathology; though NFT formation is more pronounced in mice co-expressing mutant APP and tau (Lewis et al., 2001; Hurtado et al., 2010), a similar profile is observed in mice expressing both mutant APP and wild type (WT) tau (Umeda et al., 2014). The knockdown of tau in APP/PS1 mice also eliminated AD-like pathology and prevented cognitive impairment (Leroy et al., 2012). Notably, Aβ is speculated to activate tau kinases GSK3β, CDK-5 and MAPK, leading to tau hyperphosphorylation (Takashima et al., 1996, 1998a; Ferreira et al., 1997) causing an increase in free soluble tau, which may be a critical component of Aβ induced neurotoxicity. In a *Drosophila* model of AD, administration of lithium (a GSK3β inhibitor) significantly reduced the effects of Aβ toxicity, decreasing tau phosphorylation and neuronal dysfunction (Folwell et al., 2010). Tau may also be necessary for Aβ-induced neurotoxicity; in transgenic mice expressing human APP, tau suppression resulted in an inhibition of Aβ production, and associated neuronal dysfunction (Roberson et al., 2007). Tau is also implicated in axonal transport deficits induced by Aβ via activation of GSK3β, impairing tau function and facilitating aberrant neuronal activity (Vossel et al., 2015). Further, in primary neurons from transgenic mice expressing familial AD-linked forms of human APP, suppression of tau abolished Aβ-induced GSK3β activation and thereby inhibited transport deficits (Vossel et al., 2015). The ApoE4 protein is a major genetic risk factor for AD and is associated with both senile plaques and NFTs in AD (Strittmatter et al., 1993). A recent study has demonstrated a significant effect of ApoE4 on tau-mediated neurodegeneration; in mice expressing the P301S tau mutation and ApoE4, a more severe phenotype and pathology was observed in an age dependent manner, compared to P301S mice with ApoE4 knocked down (Shi et al., 2017). In this model, the mislocalization of tau was evident as early as 3 months of age, a process which occurs through abnormal tau hyperphosphorylation. In another model expressing human ApoE4, phosphorylated levels of tau are found to correlate with ApoE4 in regions vulnerable to neurodegeneration, such as the hippocampus and cortex (Brecht et al., 2004). The interaction between ApoE4 and tau may arise through the ability of ApoE4 to modulate tau phosphorylation (Tesseur et al., 2000; Huang et al., 2001; Brecht et al., 2004). Transfection of ApoE4 in neural stem cells causes demethylation of the catalytic subunit of PP2A resulting in PP2A inactivation. Furthermore, ApoE4 is reported to modulate PP2A at the transcriptional level by regulating PP2A expression levels. These changes were also observed in post-mortem AD brain samples (Korwek et al., 2009; Theendakara et al., 2017). Thus, ApoE4 may drive tau hyperphosphorylation in familial AD, by modulating the regulatory pathways of tau and exacerbating tau-mediated neurodegeneration. In PD, α-synuclein is a pre-synaptic soluble protein that is strongly linked to pathology and is found to co-localize with tau in LBs (Arima et al., 1999; Ishizawa et al., 2003). Tau hyperphosphorylation is reported to be induced by PKA, which is triggered by the binding of α-synuclein to tau (Jensen et al., 1999). In transgenic mouse models expressing A53T human α-syn (a point mutation linked to autosomal dominant early onset PD) and in double transgenic mouse models expressing WT α-syn and P301L mutant tau, α-syn was reported to induce tau fibrillization (Giasson et al., 2003). In a *Drosophila* model of PD, α-syn and tau augmented neurotoxicity through the disruption of cytoskeletal organization, axonal transport and aberrant synaptic organization (Roy and Jackson, 2014). These data demonstrate that tau and other key disease proteins may work together in tauopathies, contributing to disease progression.

### The Tau:Iron Interaction

The current view of tau-mediated neurodegeneration revolves around there being a loss of tau function. As outlined above, the aberrant activation of tau kinases and *MAPT* mutations appear the most causative reasons for tau dysregulation in tauopathies. However, there is a new perspective emerging speculating a pivotal role for iron in tau pathology. Elevated iron levels are found in brain regions which accumulate PHFs and NFTs; such as the cortex and hippocampus in AD (Andrasi et al., 1995; Duce et al., 2010) and the basal ganglia and brainstem in PSP (Pérez et al., 1998) and is predominately concentrated within PHFs and NFTs in AD and PSP (Good et al., 1992; Smith et al., 1997; Pérez et al., 1998). Iron exists in two oxidation states: ferrous (Fe^2+^) (redox active) and (Fe^3+^) (redox-inert state); Fe^3+^ is associated with NFTs in AD and PSP (Smith et al., 1997; Pérez et al., 1998; Yamamoto et al., 2002) and can induce the aggregation of hyperphosphorylated tau. Reducing Fe^3+^ to Fe^2+^ can reverse the aggregation of tau and solubilized tau species isolated from AD brains (Yamamoto et al., 2002). This interaction may occur via a putative iron binding motif located within the MBD of tau (Smith et al., 1997; Pérez et al., 1998)—Fe^3+^ binds strongly to His residues (as demonstrated in a series of NMR experiments, Nair et al., 2010) and modification of His residues in tau isolated from AD brain prevents the interaction between iron and tau (Smith et al., 1997). Recent electrochemical studies have reported a direct interaction using X-ray photoelectron spectroscopy and CD (Ahmadi et al., 2017). This study suggested that Fe^2+^ may mediate reversible conformational changes in tau to promote its aggregation through Thr residues, conversely Fe^3+^ interactions are less pronounced but irreversible as observed by CD. Furthermore, the hyperphosphorylation of tau may not affect the interaction between tau and Fe^3+^, but Thr phosphorylation can modulate interactions between Fe^2+^ and tau (Ahmadi et al., 2017), which may explain why previous studies have speculated no interaction between Fe and tau. As noted earlier, tau hyperphosphorylation is one of the key steps in NFT formation and it can be modulated by iron through the aberrant activation of tau kinases such as GSK3ß, CDK-5 and MAPK (Egaña et al., 2003; Lovell et al., 2004; Muñoz et al., 2006; Bautista et al., 2016). Though there is debate that this process may not be the primary cause of aggregation, as hyperphosphorylated tau is evident during animal hibernation and anesthesia-induced hypothermia (Arendt et al., 2003; Planel et al., 2007), the evidence suggests that iron may act as a co-factor for tau aggregation and the phosphorylated state of tau may induce conformational changes of the protein to mediate tau:iron interactions. Consequently, tau dysregulation has been implicated in iron dyshomeostasis, as intracellular iron accumulation has been linked to a loss of tau function (Duce et al., 2010; Lei et al., 2012). Tau-KO mice accumulate iron in the cortex, hippocampus and substantia nigra causing age-dependent neurodegeneration (Lei et al., 2012), which was the result of impaired trafficking of APP to the membrane, where it interacts with ferroportin to export iron from the cell.

## Iron Metabolism

**Figure 2:**
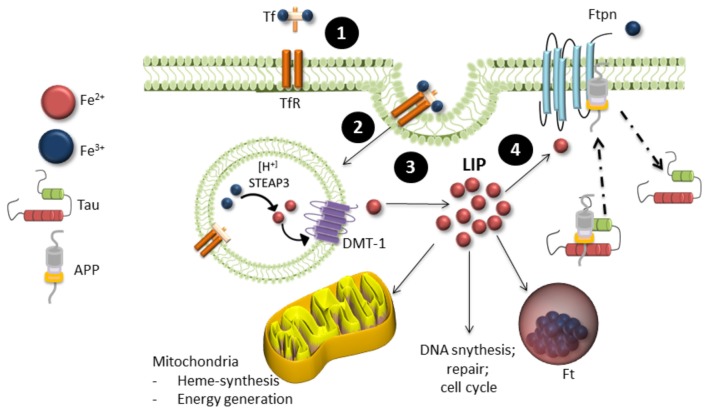
Iron metabolism in neurons. **(1)** Ferric (Fe^3+^) iron bound to transferrin (Tf) binds to transferrin receptors (TfR) on the cell surface, which undergoes **(2)**. Endocytosis. **(3)** Internalization of Tf-TfR complex causes ferric iron to be released and quickly reduced by ferric reductase (STEAP3). Ferrous iron (Fe^2+^) is then transported across the endosomal membrane into the cytosol by the divalent metal transporter 1 (DMT-1) and is released in the labile iron pool (LIP). The LIP provides a source of iron available for neuronal function, where it is either stored in ferritin (Ft) in its ferric form or proceeds to the mitochondria for biological processing. **(4)** Iron is exported from neurons by ferroportin (Ftpn), which is stabilized by an unknown mechanism involving tau, ceruloplasmin and APP. Iron is then recirculated by apo-transferrin (not shown).

Iron is the most abundant transition metal in the brain and is vital for various neuronal functions such as oxygen transport and mitochondrial respiration (Wrigglesworth and Baum, 1988; Que et al., 2008). Iron metabolism is strictly regulated, with 1–2 mg of iron absorbed and excreted from our bodies daily (Bothwell et al., 1979). Total iron homeostasis is regulated via absorption in the gastrointestinal tract and a series of protein interactions in the crypts of Lieberkühn. These proteins include a product of the hemeochromatosis gene (HFE), transferrin (Tf) one of the major iron transporters in the body, transferrin receptor (TfR) and iron regulatory proteins (IRPs). Iron exists in two oxidation states: Fe^2+^ or Fe^3+^. Fe^2+^ is highly reactive and participates in many cellular functions such as myelin synthesis, neurotransmitter synthesis and oxidative metabolism, whereas Fe^3+^ is stored in ferritin (Ft, the main iron storage protein) in its redox-inert state (Que et al., 2008). Fe^3+^ binds to Tf to form holo-Tf, which allows it to be transported through the blood brain barrier and cell membranes via transferrin receptor-mediator endocytosis (Finch and Huebers, 1982; Swaiman and Machen, 1984; Andrews, 2000). The low pH of the endosome releases Tf bound iron and it is reduced to Fe^2+^ by the six-transmembrane epithelial antigen prostate 1–4 (STEAP 1–4) and released to the labile iron pool (LIP) in the cytosol via the divalent metal transporter 1 (DMT1; Andrews, 1999; Pelizzoni et al., 2012). It is then either transported to the mitochondria to supply iron for heme and iron-sulfur biosynthesis or stored in Ft (Eisenstein, 2000). Export of iron from the cell occurs through the intramembrane protein ferroportin (Ftn). It is stabilized through several molecular interactions that includes tau, ceruloplasmin (Cp) and APP (Figure 2). Iron homeostasis is tightly regulated by IRPs, which act as the cells iron sensor. IRPs bind to iron-response elements (IREs) located in the stem-loop structures on 3′ or 5′ of untranslated mRNA of proteins involved in iron metabolism such as those mentioned above. In cases of low cellular iron, IREs bind to the 3′ end of DMT1 and TfR to prevent degradation of the proteins and increase iron levels and bind to the 5′ end of Ft to prevent Ft synthesis. When iron is abundant in the cell, iron binds to IRPs and thereby causing the degradation of TfR and DMT1 and allowing the expression of Ft.

### Iron in Neurodegeneration

Iron distribution in the brain is heterogeneous and certain regions such as the extrapyramidal regions (basal ganglia, substantia nigra and red nucleus) tend to accumulate more iron compared to other regions (Hallgren and Sourander, 1958). In the normal aging process iron accumulates predominantly in glial cells in the cortex, cerebellum, basal ganglia, substantia nigra, hippocampus and amygdala (Connor et al., 1992; Bartzokis et al., 2007). As such, brain iron accumulation is generally accepted to be a function of age, but whether it is the primary cause of neurodegenerative disorders or a result of brain degeneration is unknown. The role of iron in neurodegeneration is well documented in a class of disorders referred to as Neurodegeneration with Brain Iron Accumulation (NBIA), characterized by excess iron deposits in the extrapyramidal regions of the brain (Schneider and Bhatia, 2013). Interestingly, tau pathology is evident in the cortex, substantia nigra and globus pallidus of several NBIA cases (Arber et al., 2016), thereby demonstrating a causal link between iron dysregulation and tau. In tauopathies, several iron-associated proteins are reported to be augmented such a DMT-1, TfR and Ft. In PD, DMT-1 is upregulated in dopaminergic neurons in the substantia nigra (Qian and Wang, 1998) and increased Ft levels correlate with elevated iron levels in PSP and HD in postmortem brains (Dexter et al., 1992). In PSP and AD, tangles reportedly co-localize with Ft (Grundke-Iqbal et al., 1990; Pérez et al., 1998), suggesting that iron stored in Ft may facilitate tau aggregation, as Fe^3+^ induces tau aggregation (Yamamoto et al., 2002). Indeed, the predominant source of iron in tauopathies is speculated to be in the form of hemosiderin (Foroutan et al., 2013)—an iron storage complex comprised primarily of Ft and denatured Ft (Iancu, 1992). Iron dysregulation is hypothesized to be a source of oxidative stress in tauopathies, as the interaction between NFTs and iron is reported to act as a source for reactive-oxygen species (ROS) in neurons (Smith et al., 1997; Pérez et al., 1998; Sayre et al., 2000; Foroutan et al., 2013). Furthermore, iron is reported to interact with key disease-relevant proteins such as ApoE4, APP and α-synuclein, which as mentioned previously all interact with tau. Levels of CSF ApoE4 were reported to correlate strongly with CSF Ft (which reflects brain iron levels) in AD patients (Ayton et al., 2015), both of which are elevated in AD (Earley et al., 2000; Ayton et al., 2015). Iron is reported to upregulate intracellular ApoE in primary cortical neurons treated with iron (II) sulfate (Xu et al., 2016). In addition, carriers of the ApoE4 accumulate significantly higher levels of cortical iron, compared to patients with no ApoE4 in mild cognitive impairment (van Bergen et al., 2016). Together this data suggests ApoE4 mediated neurodegeneration is associated with brain iron levels, which in turn promotes NFT formation. Iron levels can also modulate APP expression; as a metalloprotein, increased iron levels lead to an increase in the expression of APP (Rogers et al., 2002), which may facilitate Aβ formation (Bandyopadhyay et al., 2006; Cho et al., 2010) and promote tau pathology. However, iron is also involved in the cleavage of APP via IRE within the protein and is found to modulate secretase cleavage of APP (Bodovitz et al., 1995; Bandyopadhyay et al., 2006). In rat primary cortical neurons, iron treatment resulted in the cellular retention of APP, thereby generating APP products susceptible to amyloidogenic processing in tauopathies (Chen et al., 2018). In PD, α-syn, (similar to APP) contains an IRE (Friedlich et al., 2007) and may therefore be similarly regulated by pathological iron overload. The aggregation of α-syn is also induced by Fe^3+^ through a direct interaction in the C-terminal region of the protein (Castellani et al., 2000; Giasson et al., 2000) and is postulated to be a source of ROS generation observed in PD (Levin et al., 2011).

### Evidence For an Iron:Tau Interaction From Preclinical and Clinical Trials/Animal Models and Therapies

Iron chelation therapy has been effective in clinical iron overload disorders such as ß-thalassemia major reducing morbidity and mortality (Ladis et al., 2005). Thus, the use of iron chelators such as deferoxamine and deferiprone for the treatment of tauopathies is speculated to have promising outcomes for neurodegenerative disorders such as AD (Adlard and Bush, 2018). Indeed, animal models have provided valuable insights in assessing the efficacy of iron chelation therapy for tauopathies. In transgenic mice overexpressing the P301L tau mutation, intranasal administration of deferoxamine resulted in a significant decrease in GSK3β levels, though no changes in phosphorylated tau was observed (Fine et al., 2012). However, deferoxamine restored markers of oxidation and inflammation compared to age-matched controls (Fine et al., 2012). In APP/PS1 mice, deferoxamine improved cognitive function and reduced phosphorylated tau levels and the aberrant activity of GSK3β and CDK-5 (Guo et al., 2013b). In white male rabbits, AD pathology was induced through a cholesterol-enriched diet, followed by deferiprone administration. Iron chelation reportedly reduced phosphorylated tau levels and Aβ (Prasanthi et al., 2012). A more recent study in A53T mice (a model for PD), reported improved motor function following deferiprone treatment (Carboni et al., 2017). Clinical trials have gone some way to validate the results from these animal studies in demonstrating the potential benefits of iron chelation therapy for neurodegeneration. A small deferoxamine trial in AD patients was reported to slow down the clinical progression of dementia in AD over a 24-month period (Crapper McLachlan et al., 1991). The study included two sample groups: 23 patients in the non-treatment group and 25 patients treated with deferoxamine. The rate of clinical progression of AD was measured by several behavioral assessments which included the Wechsler Adult Intelligence Scale-revised, the Wechsler Memory Scale form 1, the Western Aphasia Battery and a recorded behavioral assessment conducted in the patient’s home that was used to assess daily activities such as motor skills, left/right orientation and counting money. The primary outcome from this study was that the rate of decline in non-treated patients was twice that of patients treated with deferoxamine, which was recorded as a decrease/increase in daily activities. However, a significant number of participants dropped out from the study—resulting in *n* = 14 non-treated and *n* = 21 treated patients—and many failed to complete neuropsychiatric evaluations after the baseline assessment. The principal finding then, was based upon the recorded behavioral assessment conducted in the patient’s home. Due to the small sample population and the limited neuropsychological assessments, then the implications from this trial were limited. Furthermore, the toxicity of deferoxamine was an issue, with 30% of patients developing side effects that included lethargy, anorexia, nausea and abdominal pain, which was reversed by briefly stopping deferoxamine treatment. This may be one reason why this trial was not followed up. Other reported side effects of long term deferoxamine treatment include, audiovisual impairments, hypersensitivity reactions (Bene et al., 1989) and potentially death. The authors concluded that there was a need to develop safe and effective chelators for therapeutic use. While the aim of this study was to reduce aluminum levels in AD patients, deferoxamine has the capacity to bind several metals such as zinc, copper and iron; the affinity for iron is six times higher than that of aluminum (Gotsbacher et al., 2017), therefore it is postulated that the effects seen in the study may have been a result of iron chelation. Indeed, targeting iron has been reported to have therapeutic benefits in clinical trials of PD patients (tau is also disrupted in PD and in animal models of PD). Phase I clinical trials with a delayed start paradigm of deferiprone (30 mg/kg/day) in PD patients showed significant clinical improvements. Assessment of motor indications at 6 and 12 months using the Unified PD Rate Scale (UPDRS) showed improvements in motor function in early start patients (*n* = 20) compared to delayed start patients (*n* = 19; Devos et al., 2014). Deferiprone was also found to reduce iron deposits in the substantia nigra and putamen as measured by MRI, correlating with reduced CSF-Ft levels. Importantly, this study demonstrated that deferiprone was able to target iron in a region-specific manner; MRI analysis found no alterations in iron in unaffected brain regions such as the caudate or pallidum and no adverse effects on systemic levels of metals as measured by serum levels of Ft, Tf and ceruloplasmin. Furthermore, this study demonstrated the safety and tolerability of iron chelation therapy over a long-term period, as it was conducted over 18 months. (Devos et al., 2014). A more recent Phase II clinical trial of deferiprone in PD patients reported reduced iron levels in dentate and caudate nucleus compared to placebo following deferiprone treatment (Martin-Bastida et al., 2017). However, no difference was observed in the substantia nigra or putamen at 6 months following 20 mg/kg or 30 mg/kg deferiprone compared to the placebo group. Furthermore, no statistically significant improvements in motor function as assessed by UPDRS were observed in this trial. While both Devos et al. (2014) and Martin-Bastida et al. (2017) used the same dose of deferiprone, the discrepancies between the studies may be due to the small sample size (*n* = 5–7/group) in the latter study. Evidently more research is required in understanding the utility of iron chelators in neurodegenerative disorders, though the above studies have highlighted the potential benefits of chelating iron in the clinical setting. While deferiprone is a compound that was created in 1982 (Kontoghiorghes, 1982) for the treatment of thalassaemia, FDA approval for therapeutic use of the compound was only obtained in 2011. Deferiprone and deferoxamine are both high affinity iron chelators (K_d_ ≈ 10^−35^ M; K_d_ ≈ 10^−31^ respectively; Origa et al., 2005). However unlike deferoxamine, deferiprone is relatively small in size, crosses the blood brain barrier (Baksi and Singh, 2017) and can redistribute iron to apotransferrin (Kontoghiorghe et al., 2013)—thereby preventing systemic iron loss. As mentioned previously however, potentially serious side effects can result from the use of high affinity chelators; whilst the toxicity of deferiprone is less than that of deferoxamine, clinical studies have reported joint pains and gastrointestinal symptoms in 21% of patients with thalassemia major (Sajid et al., 2009). Deferoxamine has also been reported to decrease iron absorption and its metabolites are reported to have chelation properties (Kontoghiorghes, 1990; Lee et al., 1993), which can potentially cause iron deficiency. Furthermore, high affinity chelators are required to compete for iron with endogenous proteins such as transferrin, lactoferrin and heme containing proteins (Kontoghiorghe and Kontoghiorghes, 2016). This may have implications for the pathways associated with the formation of these iron-protein complexes such as the transferrin-iron complex, which is one of the efficient antioxidant systems located in the blood stream (Kontoghiorghe et al., 2013); the affinity of deferoxamine for iron is greater than transferrin (Aisen et al., 1978) and can therefore potentially interfere with such physiological iron-protein interactions. These issues highlight the need for “moderate” and selective iron chelators, which target abnormal iron accumulation/localization. One such compound is PBT434, which is an orally bioavailable and moderate affinity iron chelator that was designed to inhibit the aggregation of alpha-synuclein and tau. This compound is currently in phase one human clinical trial for parkinsonian diseases and has been previously tested in mouse models of PD (Finkelstein et al., 2017). In the MPTP mouse model of PD, iron levels are elevated in the substantia nigra and correlate with the accumulation of aggregated α-syn. MPTP mice treated with PBT434 (30 mg/kg/day; oral gavage) had reduced iron levels in substantia nigra compared to untreated MPTP mice, in addition to increased levels of ferroportin, reduced α-syn aggregates and improved motor function (Finkelstein et al., 2017). This study demonstrates that moderate affinity iron chelators, which should confer a more favorable safety profile compared to high affinity compounds, are sufficient to produce significant positive biological effects in mouse models of neurodegeneration. Whether this translates to efficacy in human clinical trials, however, has yet to be determined.

## Conclusion

Tau dysfunction in AD, PSP and FTD-17 (and potentially other diseases) coupled with elevated iron levels appear to drive neurodegeneration. Whilst the precise relationship between iron and tau is still unclear, there is accumulating evidence supporting the existence of a tau:iron interaction. Understanding this will help elucidate the extent to which this interaction contributes to neurodegeneration. Furthermore, given the multitude of disease-specific and other critical pathways that iron can modulate, then pharmacologically targeting this abundant metal ion may represent a tractable therapeutic approach for slowing the progression of neurodegeneration in a number of different neurological disorders as well as for promoting successful brain aging.

## Author Contributions

SR wrote the manuscript and PA edited the manuscript.

## Conflict of Interest Statement

PA is a shareholder and paid consultant for Prana Biotechnology. The remaining author SR declares that the research was conducted in the absence of any commercial or financial relationships that could be construed as a potential conflict of interest.
